# RNA Sequencing of the Human Milk Fat Layer Transcriptome Reveals Distinct Gene Expression Profiles at Three Stages of Lactation

**DOI:** 10.1371/journal.pone.0067531

**Published:** 2013-07-05

**Authors:** Danielle G. Lemay, Olivia A. Ballard, Maria A. Hughes, Ardythe L. Morrow, Nelson D. Horseman, Laurie A. Nommsen-Rivers

**Affiliations:** 1 Genome Center, University of California Davis, Davis, California, United States of America; 2 Department of Pediatrics, Cincinnati Children’s Hospital Medical Center, Cincinnati, Ohio, United States of America; 3 College of Medicine, University of Cincinnati, Cincinnati, Ohio, United States of America; University of Arkansas for Medical Sciences, United States of America

## Abstract

Aware of the important benefits of human milk, most U.S. women initiate breastfeeding but difficulties with milk supply lead some to quit earlier than intended. Yet, the contribution of maternal physiology to lactation difficulties remains poorly understood. Human milk fat globules, by enveloping cell contents during their secretion into milk, are a rich source of mammary cell RNA. Here, we pair this non-invasive mRNA source with RNA-sequencing to probe the milk fat layer transcriptome during three stages of lactation: colostral, transitional, and mature milk production. The resulting transcriptomes paint an exquisite portrait of human lactation. The resulting transcriptional profiles cluster not by postpartum day, but by milk Na:K ratio, indicating that women sampled during similar postpartum time frames could be at markedly different stages of gene expression. Each stage of lactation is characterized by a dynamic range (10^5^-fold) in transcript abundances not previously observed with microarray technology. We discovered that transcripts for isoferritins and cathepsins are strikingly abundant during colostrum production, highlighting the potential importance of these proteins for neonatal health. Two transcripts, encoding β-casein (CSN2) and α-lactalbumin (LALBA), make up 45% of the total pool of mRNA in mature lactation. Genes significantly expressed across all stages of lactation are associated with making, modifying, transporting, and packaging milk proteins. Stage-specific transcripts are associated with immune defense during the colostral stage, up-regulation of the machinery needed for milk protein synthesis during the transitional stage, and the production of lipids during mature lactation. We observed strong modulation of key genes involved in lactose synthesis and insulin signaling. In particular, protein tyrosine phosphatase, receptor type, F (PTPRF) may serve as a biomarker linking insulin resistance with insufficient milk supply. This study provides the methodology and reference data set to enable future targeted research on the physiological contributors of sub-optimal lactation in humans.

## Introduction

Breastfeeding provides numerous benefits for both mother and infant [1]–[3]. The U.S. Surgeon General [4], Institute of Medicine [5], and American Academy of Pediatrics [6] all recommend that infants be exclusively breastfed for the first 6 months with continued breastfeeding for at least 1 year. Most new mothers in the U.S. initiate breastfeeding in an attempt to follow these recommendations–but there is wide variation in lactation success with 50% of mothers failing to achieve their personal breastfeeding goals [7]. While there has been progress in recognizing how hospital and workplace barriers may undermine breastfeeding [8], the contribution of maternal physiology to lactation difficulties remains poorly characterized.

Research into the biology of human lactation has been seriously impeded by the impracticality and ethical concerns of obtaining systematic samples of mammary tissue from lactating woman. However, there is an intriguing workaround–human milk secreted during lactation is a rich source of mammary epithelial cell RNA. As lipid droplets exit the mammary epithelial cell, they are enveloped by cell membrane and secreted into milk as membrane-bound globules of fat. About 3–8% of human milk fat globules contain mammary epithelial cell cytoplasmic remnants, including RNA, captured during milk fat globule formation and secretion [9]. Maningat et al. demonstrated that the microarray-generated human milk fat layer transcriptome includes genes uniquely expressed in the lactating mammary epithelial cell [10]. Their ground-breaking work established the human milk fat layer as a potential window into mammary epithelial cell gene expression during lactation without invasive tissue biopsy.

With the advent of RNA sequencing (RNA-Seq) it is now possible to characterize the gene expression of milk-producing cells with highly sensitive detail. In contrast to microarray technology, RNA-Seq can accurately quantify both very low and very high abundance transcripts, as well as detect novel transcripts [11]. Consequently, sequencing of the mRNA found in the milk fat layer is a potentially powerful tool for identifying transcriptional signatures that underlie suboptimal lactation in women. In particular, based on epidemiologic [12] and clinical [13] research findings, we sought to determine expression of insulin signaling pathway genes and whether sequencing mRNA obtained from the milk fat layer might reveal candidate biomarker genes linking whole-body insulin action to poor lactation performance.

To date, RNA-Seq of the milk fat layer has not been reported and thus the extent to which RNA-Seq offers insights into the mammary transcriptome captured from this unique source is not known. Furthermore, it is unknown if the milk fat layer yields RNA purely of mammary epithelial cell origin, or if RNA from non-mammary epithelial sources is also present in the milk fat layer obtained from colostrum, transitional, or mature human milk.

In this paper, we describe the extraction and sequencing of high-quality RNA from the human milk fat layer over three stages of milk production, demonstrating the significance of defining lactation stage based on biochemical parameters (versus postpartum day). We then characterize the RNA-Seq transcriptome responsible for the production of biochemically-defined colostrum, transitional, and mature milk in humans, including: a) demonstrating how RNA-Seq captures the broad dynamic range of gene expression within the temporal context of secretory activation and lactation; b) functional enrichments of distinct gene expression trajectories; c) differential expression of insulin signaling genes; and d) differential expression of lactose synthesis genes. We identify key candidate genes that may control rate-limiting steps during lactation and outline future work in the endeavor to discover the biological underpinnings of poor lactation performance in women attempting to breastfeed.

## Results and Discussion

### Defining Colostral, Transitional and Mature Lactation Stages

We isolated milk fat layer mRNA from 55 fresh milk samples over 48 collections (Protocol-I: n = 24 on day 2 of lactation, with follow-up collection from n = 12 at 4–6 weeks postpartum; and Protocol-II: n = 12 convenience collections; see **Methods**). Colostrum is characteristically high in sodium (>30 mmol/L) and low in potassium (<15 mmol/L) as a result of open paracellular pathways between mammary epithelial cells [14]. Milk Na:K ratio >2.0 corresponds to pre-secretory activation (colostral) milk volumes, with declining Na:K indicating greater tight junction closure and increasing milk synthesis as secretory activation progresses [14]. We therefore categorized lactation stage for milk collected on day 2 of lactation as “colostral” if Na:K ≥2.0 (N = 10) and as “transitional” if Na:K <2.0 (N = 14). We confirmed Na:K <0.6 in our milk samples collected during “mature” lactation stage (N = 24, Protocol-I follow-up+Protocol-II), which is the expected ratio in healthy lactating (non-weaning) women ≥4 weeks postpartum [15].

### Factors Affecting Milk Fat Layer RNA Quality

Because the milk fat layer is an unconventional extracellular source of mRNA and its membrane is more fragile than cell membranes [16], we examined the vulnerability of this RNA to degradation (**Table S1**). Among colostrum samples, only 27% of RNA isolates were suitable for sequencing (RIN ≥7.0 and RNA ≥10.0 ng/*u*L). In contrast, we obtained suitable RNA from 60% of transitional and 78% of mature milk fat layer samples (Chi-square analysis by stage, *p* = 0.03). Therefore, variation in RNA quality across lactation stage must be considered when interpreting gene expression results across postpartum time points. Milk sample categorization by *day postpartum* without consideration for *lactation stage* will bias the data captured toward study participants who have progressed further toward mature milk production as these milk samples will be more likely to produce fat globules with suitable mRNA quality.

We explored several methods to optimize milk RNA quality (see **Methods**). In short, our results suggest that immediate processing with a harder, quicker centrifugation (15000 g for 10 min at 4°C) yields the best quality RNA (**Table S1**).

### Colostral Milk Fat Layer Contaminated with Somatic Cells

Under the electron microscope, milk fat globules can be observed with crescents of cytosolic components, but never nuclei [9], thus the presence of nuclei in the milk fat fraction would imply contamination with intact cells. In prior work [10], milk fat fractions were not inspected for signs of contamination with intact cells. While intact cells should form a pellet when whole milk is centrifuged, some may remain trapped in the fat layer. Indeed, under the fluorescent microscope we observed nucleated cells in milk fat fractions, presumably of leukocyte origin (**Figure S1**). We tested a washing protocol to remove these nucleated cells. Regardless of washing protocol, leukocyte-specific genes were not expressed in mature milk fat layers, scantly expressed in transitional milk fat layers and robustly expressed in colostral milk fat layers (**Figure S2**). Cell markers associated with epithelial and other (non-leukocyte) lineages are also expressed in the colostral milk fat layer. The identity of the somatic cell types present during the colostral stage and their impact on our interpretation of the milk fat layer transcriptome at this stage of lactation warrants further study.

### Lactation Genes Expressed during Colostral, Transitional, and Mature Stages

#### Descriptive characteristics of RNA-sequenced samples

We now describe our findings from sequencing the RNA isolated from 12 milk fat layer samples (colostrum, N = 2; transitional, N = 4; mature, N = 6). **Table S2** summarizes the characteristics of the study participants and their samples. Briefly, postpartum timing of sample collection ranged between 41–52 hours for colostral, 39–56 hours for transitional, and 33–130 days for mature; and Na:K ratio ranged between 5.5–9.6 for colostral, 0.70–1.15 for transitional, and 0.19–0.57 for mature.

An average of 25.5 million reads per sample was mapped to the human genome (range, 14.9 to 44.7 million). Gene expression intensity was normalized to Fragments per Kilobase of transcript per Million mapped reads (FPKM) and summarized at the gene level (e.g. all alternative splice variants counted as one gene, see **Methods**) for each sample. Using a cutoff of >0.01 FPKM to define potentially meaningful gene expression, there are 14629, 14529, and 13745 unique genes expressed in colostral, transitional, and mature stages of lactation, respectively. These gene sets and their FPKM values, identified by lactation stage and processing method, are provided in **Dataset S1**. The top 20 genes expressed at each stage are summarized in **Table 1** (mature), **Table 2** (transitional) **and** **Table 3** (colostral). The dynamic range of gene expression intensities covers several orders of magnitude: mature, 0.01–18705.5 FPKM; transitional, 0.01–3832.7 FPKM; and colostral, 0.01–2511.6 FPKM.

**Table 1:** Top 20 Expressed Genes in the Milk Fat Layer of Mature Human Milk*.

	Symbol	FPKM	Gene Name	Function of encoded protein
1	CSN2	18706	Casein, beta	Major milk protein; source of bioactive peptides and amino acids; with other caseins, forms micelles to transport calcium
2	LALBA	16905	Lactalbumin, alpha	Major milk protein and subunit of lactose synthase; forms HAMLET, a bioactive peptide that kills infected cells
3	CSN1S1	2503	Casein, alpha s1	Major milk protein; source of bioactive peptides and amino acids; with other caseins, forms micelles to transport calcium
4	CSN3	2062	Casein, kappa	Major milk protein; source of bioactive peptides and amino acids; with other caseins, forms micelles to transport calcium
5	LTF	1615	Lactotransferrin	Milk protein, major iron binding protein in milk, has stage-specific anti-microbial properties
6	FTH1	1330	Ferritin, heavy polypeptide 1	Heavy subunit of ferritin, an intracellular iron binding protein
7	CSN1S2AP	1172	Casein, alpha s2-like A	Pseudogene
8	LYZ	867	Lysozyme	Milk protein with anti-microbial activity
9	SPP1	679	Secreted phosphoprotein 1	Milk protein, up-regulates interferon-gamma and IL-12
10	TMSB10	654	Thymosin, beta 10	Function undefined
11	FASN	562	Fatty acid synthase	Catalyzes the synthesis of long chain fatty acids
12	TPT1	445	Tumor protein, translationally-controlled 1	Function undefined; possible role in cell migration
13	CEL	415	Carboxyl ester lipase (Bile salt stimulated lipase)	Milk protein, digestion and absorption of lipids
14	FABP3	340	Fatty acid binding protein 3	Arrest of mammary epithelial cell growth and proliferation
15	XDH	316	Xanthine dehydrogenase	Oxidative metabolism of purines; essential for envelopment of milk fat globules
16	ACTB	249	Actin, beta	Mammary epithelial cell motility, structure, and integrity
17	CD24	224	CD24 molecule	Cell surface sialoglycoprotein
18	EEF1A1	218	Eukaryotic translation elongation factor 1, alpha 1	Subunit of elongation factor-1 complex, translation of proteins
19	PIGR	182	Polymeric immunoglobulin receptor	Binds immunoglobulins at basolateral surface of mammary epithelial cells; complex is transported across cell and secreted into milk
20	CHRDL2	176	Chordin-like 2	Possible regulator of myoblast or osteoblast differentiation or maturation. Role in mammary biology unknown.

* Source: http://www.ncbi.nlm.nih.gov/gene/.

**Table 2:** Top 20 Expressed Genes in the Milk Fat Layer of Transitional Human Milk*.

	Symbol	FPKM	Gene Name	Function of encoded protein
1	LALBA	3833	Lactalbumin, alpha	Major milk protein and subunit of lactose synthase; forms HAMLET, a bioactive peptide that kills infected cells
2	CSN2	3833	Casein, beta	Major milk protein; source of bioactive peptides and amino acids; with other caseins, forms micelles to transport calcium
3	FTH1	1727	Ferritin, heavy polypeptide 1	Heavy subunit of ferritin, an intracellular iron binding protein
4	CSN1S1	1177	Casein, alpha s1	Major milk protein; source of bioactive peptides and amino acids; with other caseins, forms micelles to transport calcium
5	LTF	763	Lactotransferrin	Milk protein, major iron binding protein in milk, has stage-specific anti-microbial properties
6	CSN3	735	Casein, kappa	Major milk protein; source of bioactive peptides and amino acids; with other caseins, forms micelles to transport calcium
7	TPT1	603	Tumor protein, translationally-controlled 1	Function unknown; may play a role in maintaining genomic integrity in response to DNA-damaging agents
8	EEF1A1	517	Eukaryotic translation elongation factor 1, alpha 1	Subunit of elongation factor-1 complex, translation of proteins
9	TMSB10	476	Thymosin, beta 10	Function unknown
10	RPL13AP5	412	Ribosomal protein L13AP5	Ribosomal protein
11	FASN	373	Fatty acid synthase	Catalyzes the synthesis of long chain fatty acids
12	RPS28	362	Ribosomal protein S28	Ribosomal protein
13	RPL19	360	Ribosomal protein L19	Ribosomal protein
14	RPL7A	353	Ribosomal protein L7A	Ribosomal protein
15	RPL3	345	Ribosomal protein L3	Ribosomal protein
16	CSN1S2AP	345	Casein, alpha s2-like A	Pseudogene
17	RPL41	322	Ribosomal protein L41	Ribosomal protein
18	EEF2	315	Eukaryotic translation elongation factor 2	Subunit of elongation factor-2 complex, translation of proteins
19	RPS2	314	Ribosomal protein S2	Ribosomal protein
20	RPLP1	308	Ribosomal protein LP1	Ribosomal protein

* Source: http://www.ncbi.nlm.nih.gov/gene/.

**Table 3:** Top 20 Expressed Genes in the Milk Fat Layer of Colostrum Human Milk*.

	Symbol	FPKM	Gene Name	Function of encoded protein
1	FTL	2512	Ferritin, light polypeptide	Light subunit of ferritin, an intracellular iron binding protein
2	CTSD	1090	Cathepsin D	Lysosomal aspartyl protease; may assist digestion of milk proteins
3	FTH1	902	Ferritin, heavy polypeptide 1	Heavy subunit of ferritin, an intracellular iron binding protein
4	CD74	705	CD74 molecule, major histocompatibility complex, class II invariant chain	Regulates antigen presentation for immune response; cell surface receptor for the cytokine macrophage migration inhibitory factor (MIF) which controls survival pathways and cell proliferation
5	APOE	591	Apolipoprotein E	Essential for the normal catabolism of triglyceride-rich lipoprotein constituents; also interacts with many immunological processes
6	PSAP	585	Prosaposin	A glycoprotein precursor for 4 cleavage products: saposins A, B, C, and D, which facilitate the catabolism of glycosphingolipids with short oligosaccharide groups
7	B2M	485	Beta-2-microglobulin	Serum protein found in association with the major histocompatibility complex class I heavy chain on the surface of nearly all nucleated cells
8	LALBA	434	Lactalbumin, alpha	Major milk protein and subunit of lactose synthase; forms HAMLET, a peptide that kills infected cells
9	CSN2	393	Casein, beta	Major milk protein; source of bioactive peptides and amino acids. With other caseins, forms micelle to transport calcium.
10	TMSB10	357	Thymosin, beta 10	Function unknown
11	ACTB	333	Actin, beta	Mammary epithelial cell motility, structure, and integrity
12	IFI30	309	Interferon, gamma-inducible protein 30	Expressed constitutively in antigen-presenting cells and induced by gamma-interferon in other cell types
13	CTSB	299	Cathepsin B	Lysosomal cysteine proteinase; an amyloid precursor protein secretase; involved in the proteolytic processing of amyloid precursor protein; may assist digestion of milk proteins
14	VIM	245	Vimentin	A member of the intermediate filament family; highly expressed in fibroblasts
15	CD68	206	CD68 molecule	Transmembrane glycoprotein that is highly expressed by human monocytes and tissue macrophages
16	APOC1	201	Apolipoprotein C1	Expressed primarily in the liver; activated when monocytes differentiate into macrophages
17	TPT1	182	Tumor protein, translationally-controlled 1	Function unknown; may play a role in maintaining genomic integrity in response to DNA-damaging agents
18	LYZ	182	Lysozyme	Milk protein with anti-microbial activity
19	GPNMB	178	Glycoprotein nmb	A type I transmembrane glycoprotein; may be involved in growth delay and reduction of metastatic potential
20	EEF1A1	177	Eukaryotic translation elongation factor 1, alpha 1	Subunit of elongation factor-1 complex; translation of proteins

* Source: http://www.ncbi.nlm.nih.gov/gene/.

RNA sequencing results are highly correlated with qPCR (see final results paragraph, **Confirmation of genes of interest).**

#### Top genes expressed during mature lactation

**Figure 1:**
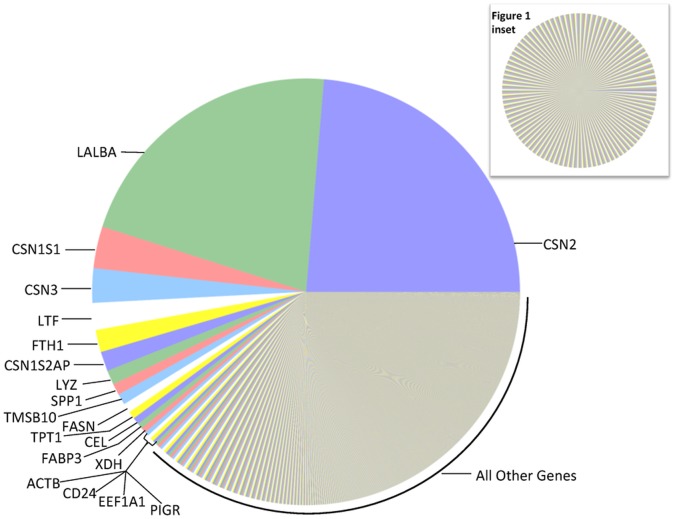
Relative mRNA abundances in the milk fat layer during mature lactation. Each pie slice represents the proportion of the total mRNA pool attributed to expression of the labeled gene. The pie slice representing the most abundant transcript occurs at the 3′o-clock position with subsequent transcripts presented counter-clockwise in decreasing order of abundance; mRNAs from the most abundant human milk proteins–CSN2 and LALBA–are ranked highest, consistent with known mammary gland biology. **Inset:** For direct comparison, we used the same approach to examine the top 500 expressed genes in the milk fat layer during mature lactation from a microarray array experiment [10]; note the markedly narrower range in relative abundances. CSN2 is ranked 18^th^ in the microarray experiment.

Stunningly, 45% of the milk fat layer mRNA during mature lactation represent just two milk protein genes: CSN2 and LALBA (**Figure 1**). Transcript abundance reflects the synthesis of proteins secreted into milk (i.e., milk *products*) *and* proteins involved in the milk making machinery (i.e., the milk making *process*). Therefore transcript abundance may not correspond closely to milk protein abundance. Furthermore, the broad dynamic range and diversity of human milk proteins has prevented accurate quantification of the human milk proteome [17]. Nevertheless, it is notable that CSN2 and LALBA encode the most abundant human milk casein (β-casein) and whey (α-lactalbumin) proteins, respectively [18]–[20]. Lactoferrin is the second most abundant whey protein in mature human milk [20], and we observed LTF to be the second most abundant non-casein gene expressed. As proteome technology progresses, future analysis of the correlation between transcript and protein abundance will be very insightful.

Given that a few abundant transcripts dominate the milk fat layer transcriptome during mature lactation, we suspected that microarray technology, in which high abundance transcripts saturate the signal, would be particularly handicapped in this biological context. Therefore, we compared our gene expression results with a supplemental data set representing the most highly expressed genes in a previous microarray study of the mature milk fat layer [10]. In the microarray experiment, the range in relative abundances of the 500 most highly expressed genes is very narrow (**Figure 1** **inset**), especially in contrast to RNA-Seq technology (**Figure 1**). Secondly, the ranking of genes by their microarray expression levels appears to be incongruent with known mammary gland biology. For example, CSN2, the most abundant human milk casein protein, appears 18^th^ in the microarray list, compared with 1^st^ in the RNA-Seq list. We conclude from the contrasting results that RNA-Seq technology provides superior insight into the milk fat layer transcriptome.

#### Top genes expressed in transitional stage of lactation

**Figure 2:**
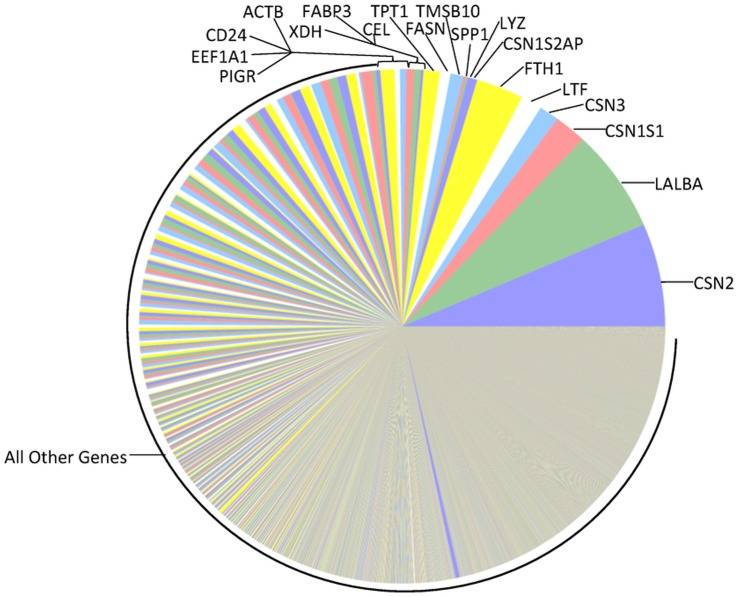
Relative mRNA abundances in the milk fat layer during transitional lactation. Each pie slice represents the proportion of the total mRNA pool attributed to expression of the labeled gene during transitional lactation. For direct comparison to Figure 1, the pie slice representing the most abundant transcript in the **Mature** transcriptome (Figure 1) is placed at the 3′o-clock position, with subsequent transcripts presented counterclockwise according to decreasing order in Figure 1. The color of the pie slice for any individual transcript is also consistent with Figure 1.

**Figure 3:**
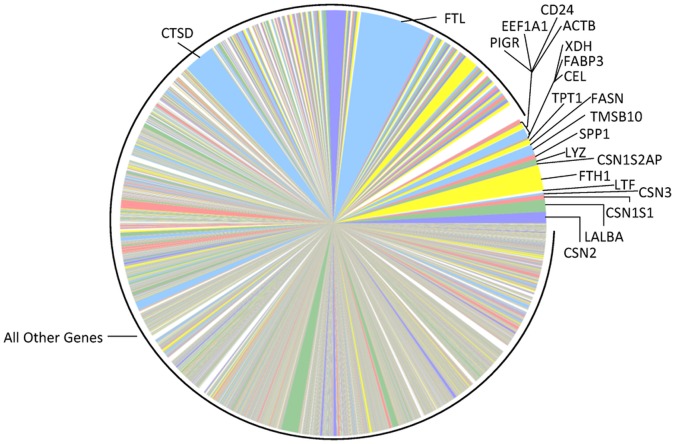
Relative mRNA abundances in the milk fat layer during the colostral stage of lactation. Each pie slice represents the proportion of the total mRNA pool attributed to expression of the labeled gene during the colostral stage. For direct comparison to Figure 1, the pie slice representing the most abundant transcript in the **Mature** transcriptome (Figure 1) is placed at the 3′o-clock position, with subsequent transcripts presented counterclockwise according to decreasing order in Figure 1. The color of the pie slice for any individual transcript is also consistent with Figure 1.

Compared to mature lactation, the milk fat layer transcriptome during the transitional stage (**Figure 2**) has similarly ranked genes (in order of abundance), but CSN2 and LALBA are not yet dominating the transcriptome. Strikingly, half of the top 20 expressed genes during the transitional stage are ribosomal proteins or translation elongation factors (**Table 2**). This stage of lactation is clearly a transition to massive protein synthesis. In general, expression of milk protein genes progresses from relatively low levels during the colostral stage (**Figure 3**), to greater dominance during the transitional stage (**Figure 2**) and strong dominance of the total mRNA pool by the most abundant milk protein genes (CSN2 and LALBA) in mature lactation (**Figure 1**).

#### Top genes expressed in colostral stage of lactation–isoferritins and enzymes

In contrast to mature lactation, the milk fat layer transcriptome during the colostral stage is predominated by transcripts for non-nutritive proteins–that is, proteins with a primary role *other than* providing amino acids to the newborn (**Figure 3**). As summarized in **Table 3**, two of the top three transcripts in the colostrum milk fat layer are isoferritins (FTL, FTH1). In 1986, Arosio, et al. reported ferritin concentration to be >50-fold higher in colostrum than in serum. Based on this finding, they hypothesized that serum was not the source of ferritin [21]. Our results, showing the isoferritins to be the top transcripts expressed in the milk fat layer of colostrum, suggest that mammary epithelial cells prioritize ferritin synthesis during the colostral stage of lactation.

The specific roles of isoferritins in neonatal health remain unclear, but here we suggest two possibilities. In other biological contexts, ferritin primarily functions as an iron-storage molecule; however, colostrum ferritin appears to have little iron associated with it [21] and thus may instead enable the neonate to sequester iron. Second, unlike the ferritin found in mature milk, colostrum ferritin is highly glycosylated [21]. These glycosylations may have an antimicrobial role, which parallels the recent finding of lactation stage-specific functional roles for lactoferrin [22]. The colostral stage is further characterized by high abundance of mRNAs (CTSD, CTSB, PSAP) that encode proteins involved in macromolecule catabolism (**Table 3**). These enzymes may be involved in the remodeling of milk-secreting cells to support the production of mature milk. However, it is also possible that they are involved in the pre-digestion of colostrum. Fragments of osteopontin in human milk have been shown to occur by cleavage with plasmin and cathepsin D [23]. A recent study of the human milk peptidome also demonstrates a natural pre-digestion of milk proteins with enzymes that originate in the mammary gland [24]. Thus, in the unique colostral stage when the neonate is not yet ready to digest proteins, it is possible that these enzymes assist with the digestion of proteins in colostrum.

To summarize, examination of the top genes expressed in our colostrum milk fat layer transcriptomes suggests that ferritins and digestive enzymes may be important components of newborn health [25] that are currently lacking for infants who do not receive mother’s milk.

### Biological Function Enrichment Analysis

The most abundant transcripts in mature milk (**Figure 1** **and** **Table 1**) are those that give rise to major milk proteins. What are the biological functions of the other expressed genes? We tested the top ten percent of the genes expressed in each stage of lactation for significant enrichment of functional annotations. Complete results are provided in **Dataset S2**.

#### Functions common across all stages of lactation

In all lactation stages, highly expressed genes are involved in mRNA translation; protein localization and transport within the cell; endocytosis; mRNA processing; protein modification; ATP biosynthesis; vesicle coating, targeting and budding; and the regulation of apoptosis. In other words, mammary genes are coordinately expressed to make, modify, package, and transport milk proteins; and to generate the ATP required for this massive protein factory.

#### Functional enrichment unique to stage of lactation

**Figure 4:**
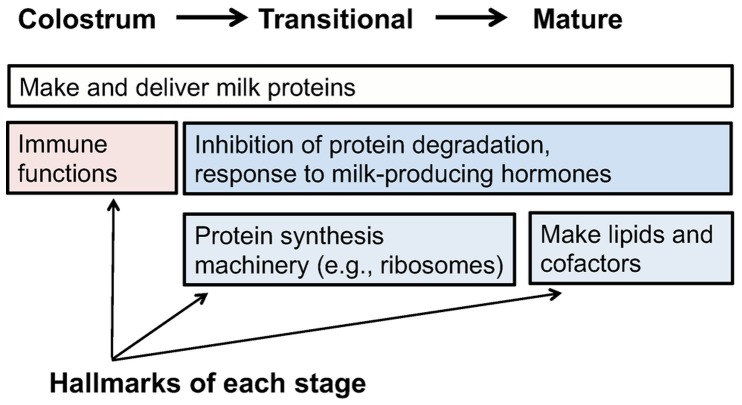
Enrichment of functional annotations for top 10% of expressing genes by stage of lactation. During all stages of lactation, protein synthesis is a significant biological function of highly expressed genes. Immune defense is a hallmark of the colostral stage. The development of the protein synthesis infrastructure and inhibition of protein degradation begins during the transitional stage. Massive lipid synthesis is a hallmark of the mature stage.

While all stages of lactation are associated with protein synthesis, some biological functions are uniquely enriched within a specific lactation stage. During the colostral stage, highly expressed genes are uniquely enriched for immune-related function, particularly “leukocyte activation.” In transitional and mature samples, highly expressed genes are associated with the inhibition of protein ubiquitination (i.e., protects proteins from degradation) and with hormone receptor binding and signaling (i.e., response to milk producing hormones). Further, lipid and lipid cofactor biosynthesis are uniquely enriched in mature samples. Consistent with our expectations, these results suggest that immune defense is a hallmark of the colostral stage, massive development of the protein synthesis infrastructure and inhibition of protein degradation is a hallmark of the transitional stage, and massive synthesis of lipids is a hallmark of the mature stage (**Figure 4**). These patterns of gene expression exquisitely reflect the function of the mammary epithelial cell during lactation and thus provides strong evidence that the milk fat layer is a valid source of mammary epithelial cell mRNA during lactation.

### Differentially Expressed Genes Across Lactation Stages

#### Cluster dendogram

Clustering analysis produced a dendogram in which lactation stage, as biochemically defined by Na:K ratio, emerges as the greatest overall difference in transcriptomes across samples (see **Figure S3**). According to the dendogram, the largest differences in the overall transcriptome occur between colostral (as defined by Na:K ratio) and all other samples. The second largest transcriptional difference occurs between transitional and mature samples. These results confirm our observation that compared to other factors such as subject, washing protocol, or time of collection, biochemically defined lactation stage (i.e., Na:K) has the largest effect on the transcriptome. Importantly, the samples did not cluster by postpartum hour or day of lactation, which is the measure typically used by lactation researchers to categorize lactation stage; our results highlight the importance of classifying samples by Na:K ratio. Consistent with this result, we observed strong inverse correlations between Na:K ratio and expression of tight junction proteins (Spearman correlation coefficients: GJB1, r = −0.84, *p* = 0.0006; CLDN3, r = −0.80, *p* = 0.002).

#### Heat map of differentially expressed genes

**Figure 5:**
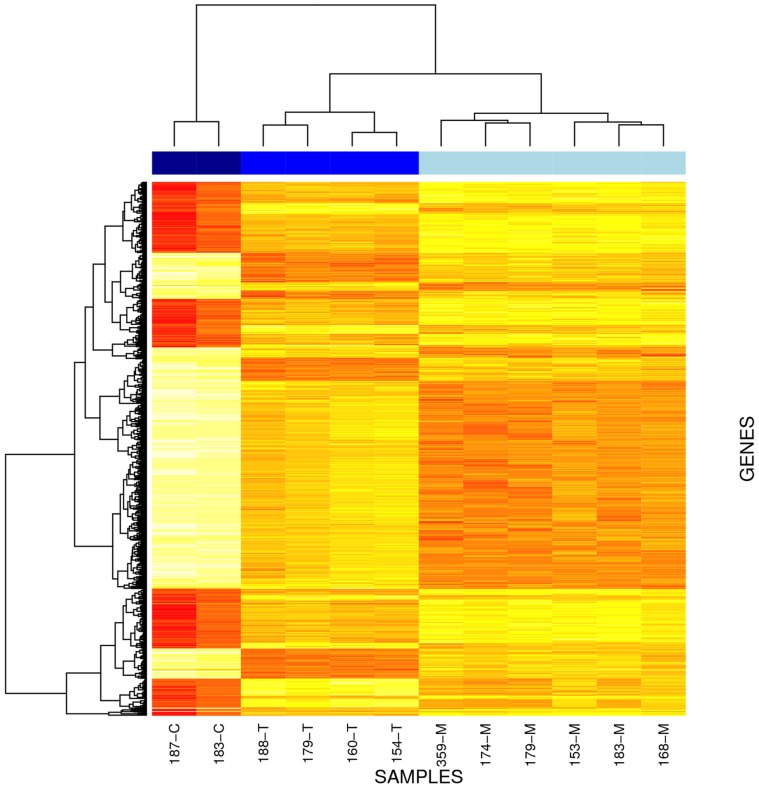
Heat map of differentially expressed genes between all stages of lactation. Columns are clustered by sample and rows are clustered by gene. Dendogram height indicates distances between clusters in gene expression profiles. The heat map illustrates lower (white/yellow) to higher (orange/red) gene expression levels with distinct transcriptional profiles across lactation stages. Blue bars at the top of each column indicate lactation stage: dark blue = colostral; blue = transitional; and light blue = mature. From left to right, starting with ID 187, postpartum timing of sample collections are 41, 52, 52, 39, 56, and 49 hours; and 130, 33, 35, 40, 24, and 45 days. Similarly, starting with ID 187, Na:K ratios are 9.6, 5.5, 0.71, 0.70, 0.98, 1.15, 0.19, 0.30, 0.57, 0.41, 0.33, and 0.45.

To test whether there was an effect of lactation stage on the expression of individual genes, we probed for differentially expressed genes using DESeq (see **Methods**). In the progression from colostral to transitional stages, 8015 genes (FPKM >0.01) are differentially expressed (3312 up-regulated, 4703 down-regulated); and in the progression from transitional to mature stages, 1948 genes are differentially expressed (939 up-regulated, 1009 down-regulated). Comparing colostral directly to mature, there are 8817 differentially expressed genes. A heat map of genes that are differentially expressed in all stages illustrates distinct transcriptional profiles by lactation stage (**Figure 5**).

#### Transcriptional trajectory analysis

**Figure 6:**
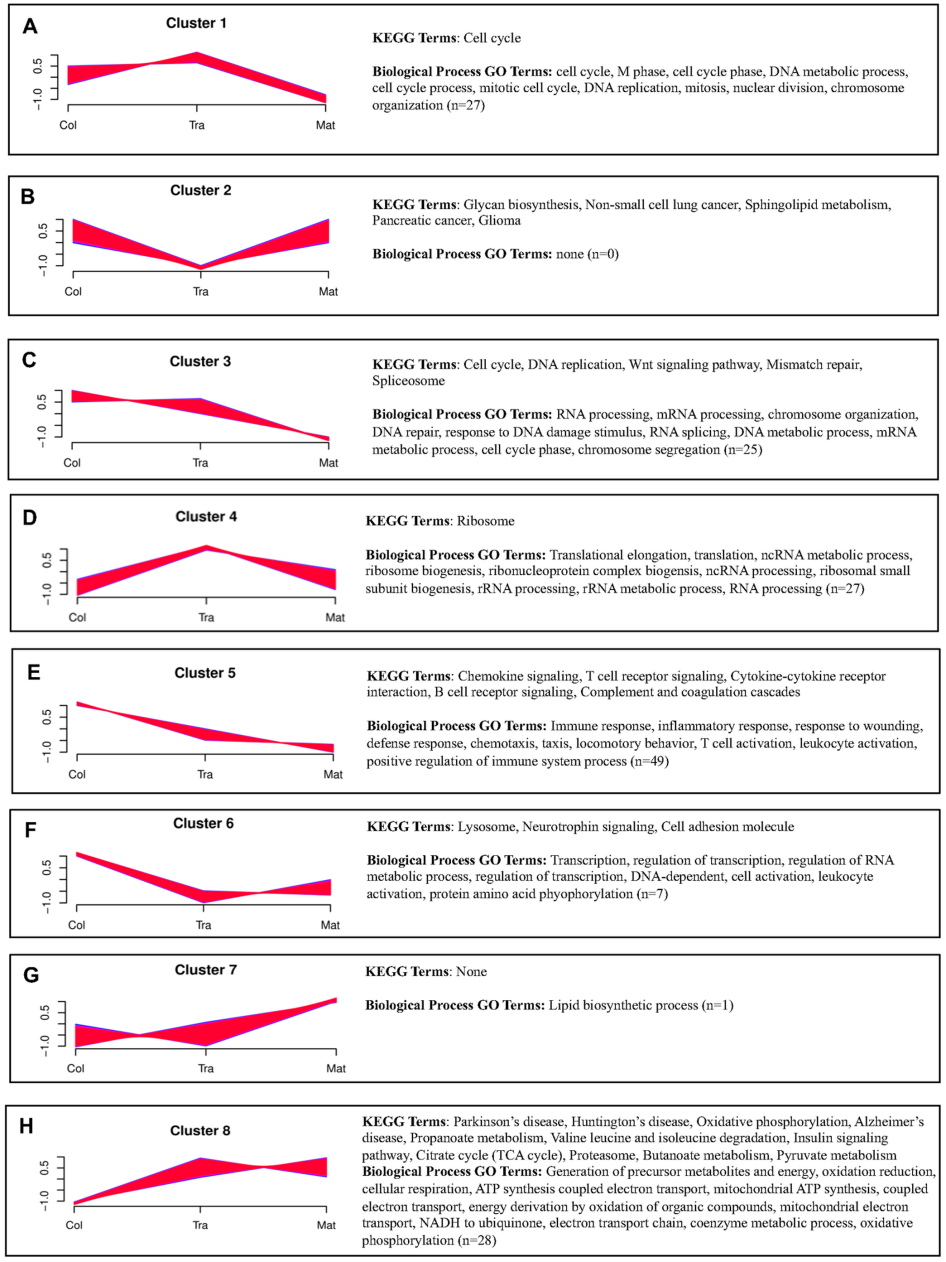
Cluster trajectories and functional enrichment across stages of lactation. **Panels A-H**: Each X-axis indicates the lactation state (colostrum, transitional, mature); Y-axis shows expression changes. Trajectory clustering analyses (see **Methods**) show that gene expression does not steadily increase or decrease in the progression from colostral to mature lactation. Eight clusters (**Panels** **A-H**) represent the major transcriptional trajectories. To the right of each cluster are shown the statistically enriched KEGG pathways and the top 10 significantly enriched biologic process GO terms (n = total number of significantly enriched biologic processes).

The heat map illustrates that not all genes are steadily increased or decreased in the progression from colostrum to mature lactation. Therefore, we conducted a clustering analysis of all expressed genes to determine the primary gene expression trajectories. We systematically repeated the clustering analysis with increasing numbers of clusters to identify the minimally redundant set with the most clusters (see **Methods**). Eight clusters optimally represent the major transcriptional trajectories (**Figure 6** **, Panels A-H**).To determine the function of genes with similar transcriptional trajectories, the gene members of the eight clusters were analyzed for overrepresented functional annotations. Significant KEGG pathways and the top ten significant Biological Process Gene Ontology Terms are shown next to each cluster in **Figure 6** (with a complete summary in **Dataset S3**).

As expected, genes associated with “cell cycle” (**Figure 6** **, Panels A and C**) and “immune response” (**Figure 6** **, Panel E**) are up-regulated in early lactation and down-regulated as lactation progresses. Likewise, “lipid biosynthesis,” which provides a major source of energy for the neonate but not immediately needed to protect the infant’s gut, does not peak until the mature stage of lactation (**Figure 6** **, Panel G**).

The remaining clusters are more novel. N-glycan biosynthesis and sphingolipid metabolism are coordinately increased during colostral and mature stages relative to transitional (**Figure 6** **, Panel B**). Infant-associated gut bacteria use a novel endoglycosidase to liberate N-glycans from glycoproteins as a unique food source for these beneficial gut bacteria [26]. It is possible that the upregulation of N-glycan biosynthesis during the colostral phase is a mechanism to establish a healthy gut microbiome. Glycosphingolipids are essential components of the milk fat globule membrane and they additionally have been found to adhere to enterotoxigenic *Escherichia coli* [27]. Thus, genes involved in the production of glycosphingolipids may be up-regulated in early lactation to protect the neonate from pathogens and again during the mature stage of lactation to produce the milk fat globule membranes that envelope secreted lipids as they reach maximum production.

The distinctly different clusters shown in **Figure 6** **-Panels D and F**, and their significant associations with translation and transcription, respectively, suggest a complex interplay of these regulators of milk synthesis. Genes associated with transcription are most abundant in the colostral samples (**Panel F**) while genes associated with translation are most abundant in the transitional samples (**Panel D**). Physiologically, the transitional stage is marked by the onset of copious milk production which requires building the infrastructure for massive milk protein synthesis that continues throughout lactation. Taken together, these trajectory enrichment results suggest that during the colostral stage genes are being expressed that will in turn activate further expression of the genes that build the ribosomal infrastructure required for large scale translation–i.e., preparation for massive milk protein synthesis has already begun. Further, gene expression during the transitional stage of lactation suggest post-transcriptional regulation of protein synthesis, with enrichment of gene ontology terms related to translation and RNA processing, especially non-coding RNA processing. These results are consistent with prior predictions that secretory activation is post-transcriptionally mediated [28], [29]. In summary, RNA-Seq provides insight across multiple levels of regulation of milk protein synthesis.

Finally, **Figure 6** **, Panel H** contains genes up-regulated in transitional samples relative to colostral samples before reaching steady-state. These genes are associated with the insulin signaling pathway and with the generation of energy. We inspect the modulation of the insulin signaling pathway in detail in the next section.

### Modulation of Insulin Signaling during Lactation

In bovine and rodent models, insulin has recently been shown to play a central role in milk protein synthesis [29]–[31]. As described in the previous section, we also observed strong modulation of insulin signaling genes in our data derived from lactating women.

#### Differential expression of insulin signaling genes between colostral and transitional stages

**Figure 7:**
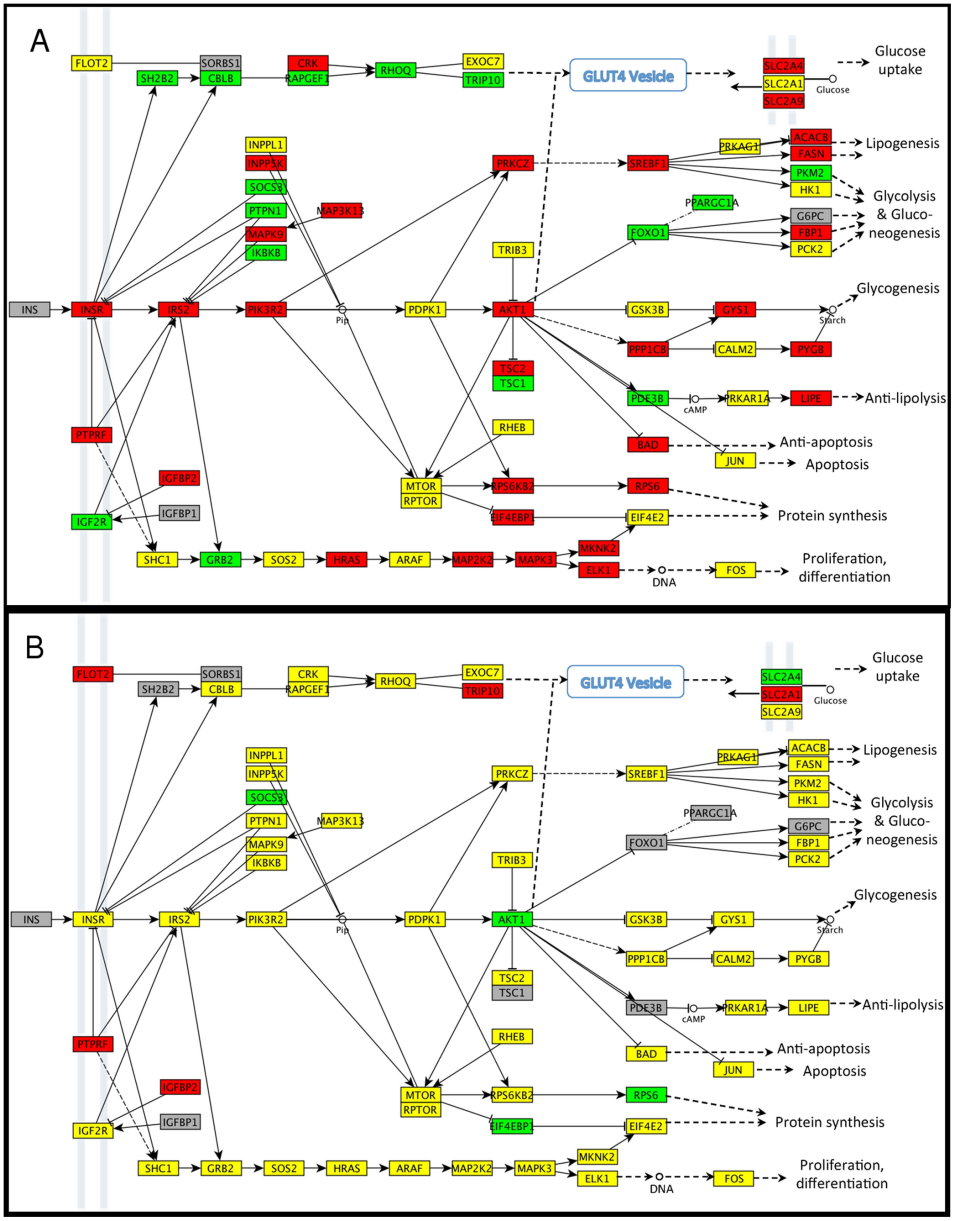
Relative expression of genes encoding proteins in the KEGG Insulin Signaling pathway. **Panel A**: Strong up-regulation of genes encoding insulin signaling proteins occurs between colostral and transitional stages; **Panel B**: signaling is attenuated between transitional and mature stages. Panels A-B: Gene symbols are detailed in Dataset S4. Color denotes change in gene expression from colostrum to transitional (Panel A) or transitional to mature (Panel B): red = significant increase, green = significant decrease, and yellow = no significant change in expression between stages; gray = no member of this gene family is expressed >0.5 FPKM during either stage being compared. Differences with *p*<0.05 after adjustment for multiple hypothesis testing (method = “BH”) were deemed statistically significant. Symbols: ↓ activation; ⊥ inhibition; –>indirect effect. Full symbol key at http://www.genome.jp/kegg/document/help_pathway.html.

Progressing from colostral to transitional lactation, among the 69 gene/gene family nodes represented on the KEGG insulin signaling pathway, 30 were up-regulated, 14 were down-regulated, and an additional 20 were robustly expressed (FPKM >0.5) but not significantly changed between colostral and transitional lactation (**Data set S4**). Overall, there is a pattern of strong modulation of insulin signaling between colostral and transitional lactation, with up-regulation of lipogenesis and protein synthesis; and inhibition of apoptosis, glycolysis, and glycogenesis (**Figure 7** **, panel A)**.

Using a murine model, Berlato and Doppler [32] elegantly demonstrated that gene expression for insulin receptor isoform A (INSR-A), IGF1 and IGF2 receptors (all of which activate cell growth pathways [33]) predominate in the mammary gland during early pregnancy; then during lactation IGF2 receptor (IGF2R) declines 5-fold and INSR-B (which activates cell metabolism pathways [33]) becomes more predominant. We did not sequence at a depth that allows us to separately quantify expression levels of the two insulin receptor isoforms, but our pattern of insulin signaling is consistent with the aforementioned study. For example, in progressing from colostral to transitional lactation, INSR increases 3.6-fold, IGF2R declines 3.7-fold, and IGF1R is not being expressed at all. Further, IGF2BP2, a known suppressor of IGF2 signaling, is upregulated 22-fold between colostral and transitional lactation (and further increases 5.3-fold between transitional and mature lactation).

#### Differential expression of insulin signaling genes between transitional and mature stages

Further progressing from transitional to mature lactation, 5 gene/gene family nodes in the insulin signaling pathway were upregulated, 5 were downregulated, and an additional 49 were robustly expressed (FPKM >0.5), but not significantly changed between transitional and mature lactation (**Data set S4**). Overall, insulin signaling predominantly maintains steady-state robust expression between transitional and mature lactation with some up-regulation of vesicle trafficking (indicated by increased FLOT2 and TRIP10 expression) and some toning down of the steep up-regulation of metabolic signals during transitional lactation (indicated by significant down-regulation of AKT1, RPS6, and EIF4EBP1; and up-regulation of PTPRF). Of note, PTPRF (protein tyrosine phosphatase, receptor type F, also known as LAR) suppresses insulin action by dephosphorylating insulin receptor substrate (IRS) proteins, thus modulating systemic insulin signals to tissue-specific needs [34]. Breast engorgement peaks at around days 4–5 postpartum [35] followed by down-regulation of milk synthesis to a rate commensurate with milk removal. Our results suggest that PTPRF could be part of an orchestrated “fine tuning” of milk synthesis rate.

#### PTPRF may be overexpressed in insulin resistant mothers

Referring back to the heat map shown in **Figure 5**, it is notable that among the mature samples, two main clusters emerged, which we will refer to as: Mature Group 1 (ID 359, 174, and 179) and Mature Group 2 (ID 153, 183, and 168). Interestingly, where available, these groups exhibit distinct clinical characteristics (see **Table S2**). First, in Mature Group 2, maternal report of the onset of notably fuller breasts occurred nearly two days later than in Mature Group 1 (median, 74 versus 34 hours, respectively). Second, in relation to the larger follow-up cohort (N = 12, see 1^st^ paragraph of results), women in Mature Group 2 were below the median in both insulin secretion and insulin sensitivity, whereas women in Mature Group 1 were above the median for these measures. Third, women in Mature Group 2 all reported difficulty with their milk supply either during the day 4 postpartum interview and/or at the 4–6 week postpartum follow-up interview. Therefore, we questioned whether PTPRF gene expression might be elevated in Mature Group 2 samples, as PTPRF overexpression could be causing excessive downward modulation of the insulin signaling pathway in the women with lower insulin sensitivity. In fact, we did observe significantly higher expression of PTPRF in Mature Group 2 versus Group 1 samples (9.1±0.7 versus 7.5±0.7, *p* = 0.047). In contrast, we found no difference between these two groups in prolactin-sensitive STAT gene expression (STAT3, STAT5A, STAT5B, all *p*>0.67). This novel finding is consistent with evidence implicating PTPRF overexpression as a contributor to insulin resistance in other organ systems [34], [36] and in the pathogenesis of Type II diabetes [37]. Based on these initial findings, we hypothesize that women with decreased insulin sensitivity will experience a more sluggish increase in milk output in response to infant demand as a result of PTPRF overexpression in the mammary gland. Granted, our hypothesis is based on a small number of samples and more investigation is warranted before drawing firm conclusions. Therefore, we plan to follow up this very preliminary, but biologically plausible, hypothesis in a larger study designed specifically to examine the relation between insulin resistance, PTPRF expression and mammary gland responsiveness.

### Lactose Synthesis Pathway

**Figure 8:**
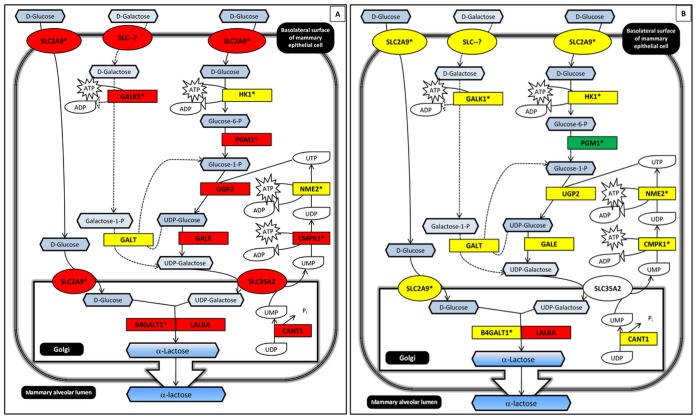
Relative expression of genes encoding enzymes in the lactose synthesis pathway. The rate of lactose synthesis is an important driver of milk production level. **Panel A**: Strong up-regulation of genes encoding lactose synthesis enzymes occurs between colostral and transitional stages; **Panel B**: UDP-Glucose production (PGM) is attenuated between transitional and mature stages. Notably, this occurs while LALBA continues to increase in expression. Panels A-B: Gene symbols are detailed in Dataset S4. Color denotes change in gene expression from colostrum to transitional (Panel A) or transitional to mature (Panel B): red = significant increase; green = significant decrease; and yellow = no significant change in gene express levels between stages. Differences with *p*<0.05 after adjustment for multiple hypothesis testing (method = “BH”) were deemed statistically significant. Footnotes: * more than one member of this gene family is expressed >0.5 FPKM; **–** Alternate pathway for producing UDP-Galactose; **SLC–?**, specific family member that translocates D-Galactose is not known.

The rate of lactose synthesis is a key driver of milk production; however, “lactose synthesis” is not an annotated pathway. We manually constructed this pathway and overlaid our gene expression results (**Figure 8**). We observed strong up-regulation of nearly every gene in this pathway between colostral and transitional stages (**Figure 8** **, Panel A**) and robust steady-state expression, with significant attenuation of PGM1 (*p* = 0.02), between transitional and mature lactation stages (**Figure 8** **, Panel B**).

The most up-regulated and expressed gene in the lactose synthesis pathway is LALBA, increasing 10.2-fold from colostral to transitional lactation and 6.1-fold from transitional to mature lactation. This is to be expected given that in addition to being a member of the enzyme complex involved in the final step of lactose synthesis, LALBA is also the predominant whey protein in human milk.

#### Glucose transport into the mammary epithelial cell

Glucose is an important fuel source for the synthesis of lactose. The solute carrier family 2A genes (SLC2A, GLUT protein family) are involved in glucose (and other solute) transport across cell membranes. We detected expression of several SLC2A genes in the milk fat layer of mature samples (SLC2A1, 4, 5, 6, 8, 9, 10, 11, and 13). SLC2A1 is thought to be the primary route for glucose entry into the lactocyte across species based on reports from rodent and bovine studies [38], [39]. Curiously, we observed SLC2A9 to be the most strongly up-regulated across lactation stage and the most abundantly expressed in mature samples. SLC2A9 increased from 1.9 to 4.0 to 5.7 FPKM across colostral, transitional and mature lactation stages, respectively. Little is published on SLC2A9 in the mammary gland, but our data suggest this member of the SLC2A family deserves further attention, including whether it is involved in glucose and/or transport of other solutes into the mammary gland.

#### Rate-limiting enzymes of lactose synthesis

There are two potential sources of UDP-Galactose in the mammary epithelial Golgi, either through the uptake and metabolism of D-Galactose or D-Glucose. Our results show that both pathways are up-regulated between colostral and transitional stages. However, the more predominant route appears to originate with D-Glucose, as the enzymes unique to this pathway are up-regulated 5.9-fold (PGM1) and 8.2-fold (UGP2). In contrast, GALK1 is up-regulated 3.3-fold.

Similar to what we observed for the insulin signaling pathway, the strong up-regulation of the lactose synthesis pathway observed between colostral and transitional lactation stages is attenuated between transitional and mature lactation stages. Gene expression for the enzymes central to the synthesis of UDP-Glucose from D-Glucose (PGM1, UGP2, and NME2) are down-regulated, with only PGM1 attaining statistical significance (0.58 fold-change, mature vs. transitional, *p* = 0.02), suggesting these nodes are critical to the regulation of lactose synthesis. SLC2A9, SLC2A1 and SLC2A4RG are all modestly up-regulated (only SLC2A1 significant, increasing 2.0-fold) and LALBA is further up-regulated 6.1-fold, consistent with increased glucose utilization and milk protein output in mature lactation. All other lactose synthesis genes are expressed at a level similar to transitional samples. While protein abundances are often not linearly reflected by mRNA levels, these transcriptional results do suggest key network nodes to probe in future studies. In summary, we hypothesize that the rate-limiting enzyme PGM1 modulates milk yield in humans.

### Confirmation of Genes of Interest

**Figure 9:**
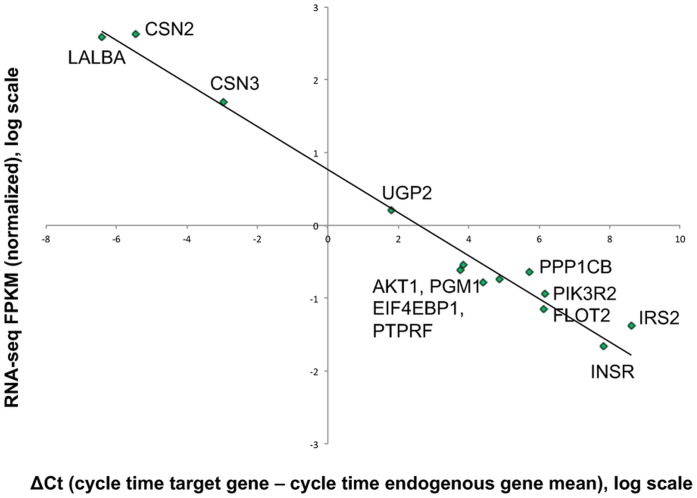
Confirmation of RNA-Seq results with qPCR results. Data derived from average of 4 milk fat layer samples obtained during mature lactation. Results are plotted on a log scale for both axes. X-axis: qPCR ΔCt (target gene, cycle threshold - geometric mean of endogenous genes, cycle threshold); Y-axis: RNA-seq FPKM (target gene FPKM/geometric mean of endogenous genes FPKM). Endogenous genes selected: ACTB, RPS18, SSH3. Correlation of gene expression measured by qPCR versus RNA-seq, R^2^ = 0.98, *p*<0.001.

To confirm our RNA-Seq findings using a different assay, we probed the following genes of interest in 4 samples from mature lactation using qPCR: the major milk protein genes (LALBA, CSN2), the gene most abundant in the mature milk fat layer transcriptome using microarray technology (CSN3, from Maningat et al. [10]), and a selection of key genes differentially expressed in the lactose synthesis pathway (PGM1 and UGP2) and insulin signaling pathway (INSR, IRS2, AKT1, EIF4EBP1, PPP1CB, PIK3R2, FLOT2, and PTPRF). Details on the primer probes sets for these genes are provided in **Dataset S5**. The normalized mean ΔCt was calculated across the four mature samples for each gene (see **Methods**). The linear relation (on a log scale) between normalized RNA-Seq FPKM and qPCR ΔCt across all target genes is very strong (**Figure 9**, R^2^ = 0.98, *p*<0.001), even though the mean expression of these genes varies by 5 orders of magnitude (10^−1^ to 10^4^ FPKM). These PCR results confirm our RNA-Seq findings.

### Conclusions

Non-invasive sampling of the transcriptome of milk-producing cells via RNA secreted into human milk provides a powerful window into the biology of human lactation. In this paper, we explored several methodological issues in using human milk fat layer RNA for this purpose. We found that: a) lactation stage must be defined biochemically (such as Na:K ratio), not by day of lactation, b) lactation stage influences RNA quality, quantity, and potential immune cell contamination, c) immediate processing and hard, fast centrifugation yield better quality RNA, and d) additional washing of the milk fat layer is not necessary for the analysis of mature milk samples and does not decrease immune cell contamination in colostrum samples. We also found that RNA sequencing yields superior results compared to microarray experiments.

Our RNA-Seq results paint the most vivid portrait to date of the transcriptome responsible for human milk production. Strikingly, transcripts of β-casein and α-lactalbumin genes make up 45% of the total pool of mRNA during mature lactation. Consistent with known physiology, genes expressed during lactation are associated with making, modifying, transporting, and packaging these highly abundant milk proteins. Lactation-stage specific transcripts are associated with immune defense during the colostral stage, the up-regulation of massive milk protein synthesis during the transitional stage, and the full-scale production lipids and other factors during the mature stage of lactation. Furthermore, fundamentally different regulatory mechanisms appear to control milk production during the different stages of lactation. First, genes whose products participate in transcription and translation are differentially regulated across all three stages. Second, we see dramatic changes in the transcriptome even after the onset of copious milk production (e.g., transitional versus mature stage). Future experiments, utilizing the methods outlined in this paper, are needed to determine the comprehensive acute, long-term, local, and systemic mechanisms by which women produce milk in response to infant feeding patterns. For example, the effect of early milking frequency on “programming” milk production capacity has been explored in bovine models [30], [40]–[42], but no human studies have been conducted, most notably due to the previous need for mammary biopsies.

Correlates of impaired insulin and glucose metabolism have recently been reported to be associated with poor lactation performance in women [12]. In the current study, we found that the transcriptomes of mature samples clustered by measures of maternal insulin secretion and sensitivity. While our study is limited by sample size and indirect measures of insulin and glucose metabolism, these tantalizing data provide motivation to design future studies to specifically test hypotheses regarding maternal insulin action and lactation performance.

In summary, we have presented the essential methodology, reference data set, and preliminary results derived from the RNA-Seq analysis of the human milk fat layer transcriptome across three stages of lactation. Our findings set the stage for future research on the biological contributors to poor lactation performance in women.

## Materials and Methods

### Participants

Fresh milk was collected from participants enrolled in one of two research protocols.

#### Ethics statement

All participants provided written informed consent. **Protocol-I** was approved by both Cincinnati Children’s Hospital and The Christ Hospital (maternity hospital) Institutional Review Boards. The *ad hoc* milk samples described in **Protocol-II** were obtained through an ongoing Research Human Milk Bank protocol [43] approved by the Cincinnati Children’s Hospital Institutional Review Board.

#### Protocol-I, Day 2 of Lactation

Participants were enrolled at a Cincinnati-area maternity hospital during their postpartum stay. On each recruiting morning (24 days between June 9– September 9, 2011), a maternity nurse identified mothers who were between 36–60 hours postpartum. Proceeding from a randomly ordered list, the nurse explained the study and screened mothers for the following eligibility criteria: English-speaking, currently breastfeeding, aged >18 years, without history of major breast surgery, healthy singleton infant, and telephone access for follow-up contact. Upon obtaining written informed consent, the nurse administered a breastfeeding assessment and then guided the study participant in collecting 2.5 mL of milk from each breast by mechanical breast expression using a sterile collection shield and container (**Day 2 milk**). A study research assistant noted the exact time of collection and transported the milk on ice to the lab for immediate processing. Mothers were telephoned on the fourth postpartum day (between 72–96 hours) to collect socio-demographic data and inquire about timing of onset of noticeable breast fullness, which is a validated indicator of secretory activation (the onset of copious milk production, previously referred to as stage II lactogenesis) [44], [45]. Early postpartum body mass index (kg/m^2^) was estimated as: ((maternal weight as recorded upon admission to the labor and delivery unit) – (2×birth weight))/admission height^2^).

#### Protocol-I, Follow-up

At 4–6 weeks postpartum, Protocol-I participants who were still lactating and not pregnant were asked to provide a follow-up milk sample during a morning visit to the Cincinnati Children’s Hospital Clinical Research Center. A 10 mL aliquot of fresh milk (**mature milk**) was collected from a complete expression of the left side at least two hours since the previous feeding. The participant underwent an oral glucose tolerance test and completed an infant feeding practices interview during the same morning visit.

#### Protocol-II

Women were recruited through posted announcements. After written informed consent, these participants provided a single, 10 mL aliquot of freshly expressed milk (**mature milk**). Maternal age, ethnicity, day of lactation, and breastfeeding exclusivity were recorded at the time of collection.

### Milk Fat Globule Processing and Detection of Intact Cells

Fresh milk was aliquoted into three 2.0 mL tubes and spun in an Eppendorf MicroCentrifuge 5417R. After centrifugation of fresh milk, fat globule-enveloped mammary epithelial cell RNA will reside in the fat layer at the top of the tube and–presumably–extraneous RNA from intact cells (leukocytes, sloughed epithelial cells and stem cells) will reside in the cell pellet at the bottom of the tube, effectively separating the two sources of RNA [16]. We tested this presumption by examining the fat layer for nuclei (as a marker of intact cell presence). To accomplish this, the fat layer solids were fixed on a slide with a cytospin, stained with DAPI, and examined for blue-fluorescing nuclei on a Zeiss florescent microscope. As we attempted to reduce intact cells in the fat layer, the centrifuge speed and processing method evolved over 3 phases (soft spin, hard spin, washed), as described below.

#### Soft spin

We followed the method described by Maningat [10]. After a soft spin (300 g for 10 min at 4oC), the milk fat layers from three, 2.0 mL aliquots were transferred into a single new tube to which 500 µL TRIzol® was added and stored at −80°C. In a subset of samples, ∼1 µL was removed prior to adding TRIzol®, to examine for evidence of intact cells (described in above paragraph). Colostrum milk fat fractions were heavily infiltrated with intact cells (**Figure S1, Panel A**). Mature milk fat fractions, although to a lesser extent, were also infiltrated with intact cells (**Figure S1, Panel B**). Thus, we proceeded to a hard spin.

#### Hard spin

After a hard spin (15,000 g at 4°C), the milk fat layers were processed as in the soft spin protocol, above. The hard spin did not reduce RNA quality (see RNA isolation section, below), but it also did not reduce infiltration of the fat layer with intact cells, which were evident to the same degree as after a soft spin (not shown). We then proceeded to a milk fat washing protocol.

#### Washed

In this phase, milk fat layers were washed as follows. After a hard spin at a warmer temperature, (15,000 g at 12°C) the milk fat layers were transferred to a clean set of 3 tubes and re-suspended in 1 mL PBS+10 µM EDTA. After centrifuging again (15,000 g at 12°C), the milk fat layers from all 3 tubes were transferred to a single tube, re-suspended in 1 mL PBS, and centrifuged a final time (15,000 g at 12°C). The remaining milk fat layer was transferred into a single new tube to which 500 µL TRIzol® was added and stored at −80°C.

Under Zeiss florescent microscope, the washed method appeared to reduce the number of intact cells in colostrum samples and eliminate them from mature samples, but to definitively determine if washing was beneficial in removing infiltration of intact cell RNA, we sequenced both hard and washed milk fat samples. As part of this approach, some milk samples were split so that an aliquot was processed using the hard method and another using the washed method. We conducted both a targeted comparison of gene expression for immune cell markers (such as CD45, a cell surface marker unique to immune cells) and a global examination of differential gene expression by washing method.

### Milk Sodium and Potassium Analysis

Milk sodium concentration is a marker of tight junction closure between mammary epithelial cells; with the onset of secretory activation (stage II lactogenesis) milk sodium concentrations decrease sharply as potassium concentrations increase [14]. After the initial spin of the fresh milk sample we transferred the aqueous fraction to a second tube and stored at −80°C. We thawed and assayed batches of 8 in duplicate for sodium and potassium concentration using flame photometry (Cole-Parmer Dual-Channel Flame Photometer, Vernon Hills, IL). Within each sample, we calculated Na:K from duplicate runs (mean of [sodium_1_/potassium_1_], [sodium_2_/potassium_2_]). Expression of sodium as a ratio to potassium adjusts for slight variations in lipid-free purity of the aqueous fraction [15].

### Maternal Glucose and Insulin Sampling and Analysis

Among the subset of participants in Protocol-I who agreed to a follow up visit at 4–6 weeks postpartum, we conducted an oral glucose tolerance test at the time of collecting a follow up milk sample. For this, participants arrived at our clinical research center after an 8–10 hour fast. We obtained blood samples at 0, 30, 60, 90 and 120 minutes after the participant consumed a standard 75 g glucose beverage. The samples were assayed for serum glucose (glucose oxidase method) and insulin (electro-chemi-luminescence immunoassay) concentrations at the Biochemistry Core Lab at Cincinnati Children’s Hospital. The results were used to calculate maternal insulin sensitivity (ISOGTT) [46] and oral disposition index (ISSI-2, a measure of pancreatic beta-cell function) following the method described by Renakaran [47].

### RNA Isolation, Quality Evaluation and Selection for Sequencing and Analysis

For all lipid samples we extracted and purified total RNA in batches of 16 using the PROMEGA Maxwell 16™ integrated system (Promega Corporation, Madison, WI). We assayed a 2 µL aliquot for RNA concentration, purity (rRNA 28 s∶18 s ratio), and quality (RNA Integrity Number (RIN)) using the Agilent 2100 Bioanalyzer (Santa Clara, CA).

We initially selected 16 purified RNA samples for paired-end sequencing based on these priorities: RIN ≥7.0 and RNA ≥10.0 ng/uL; paired samples across time points (Day 2 and mature) and split between washed and unwashed. In the Day 2 samples, sodium and potassium concentrations ranged between 6.2–90.9 mmol/L and 9.5–20.5 mmol/L, respectively; and Na:K ratio ranged between 0.42 (greater tight junction closure between mammary epithelial cells) to 9.62 (less tight junction closure). Only three Day 2 samples categorized as colostral stage were suitable for sequencing (RIN ≥7.0 and RNA >9.0 ng/uL) and all three were selected.

All gene expression results subsequent to washing protocol comparisons are based on a final set of 12 sequenced samples (N = 2 colostral, N = 4 transitional, and N = 6 mature) remaining after the exclusion of the unwashed partner in the split pairs (n = 1 colostral, n = 2 mature) and the exclusion of 1 transitional sample from a mastitic participant (ID = 158). **Table S2** summarizes maternal and sample characteristics for sequenced samples.

### RNA Sequencing Protocol and Computational Pipeline

From the samples we selected for sequencing, the Gene Discovery core facility at Cincinnati Children’s Hospital prepared RNA-Seq libraries using the Illumina TruSeq RNA kit (Illumina, Inc., San Diego, CA) and performed sequencing on an Illumina HiSeq2000 with version 3 chemistry. Libraries were multiplexed in batches of 12 to ensure a minimum of 20 million 100 bp paired-end reads per sample. FASTQ files were de-multiplexed to assign reads to the originating sample. The data has been deposited in NCBI’s Gene Expression Omnibus and are accessible through GEO Series accession number GSE45669 (http://www.ncbi.nlm.nih.gov/geo/query/acc.cgi?acc=GSE45669).

Reads were mapped to the human genome assembly hg19 using TopHat [48] and NCBI’s Reference Sequence annotations. Transcript abundances were estimated as FPKMs (fragments per kilobase of exon per million fragments mapped) using Cufflinks [49]. Genes with a mean expression level less than 0.01 FPKM across all time points and all samples were excluded from further analysis.

To facilitate other types of analyses, counts for each hg19 Ensembl gene were calculated by applying HTSeq-count (http://www-huber.embl.de/users/anders/HTSeq) to the mapped reads (e.g. TopHat output) after preparation with Samtools and Perl to identify read pairs and remove those with mates on different chromosomes. The hg19 Ensembl gene reference annotation was derived from Ensembl Release 62 [50]. HTSeq-count was run in “intersection-nonempty” mode.

To conduct clustering analyses, an R package, DESeq [51], was used to apply a variance stabilizing transformation to the count data. Samples were then clustered in R by Euclidean distance of the variance-stabilized count data. Gene expression profiles were clustered in R using the mfuzz package [52], a “soft” clustering algorithm which allows genes to be assigned to more than one cluster and quantifies the degree to which the cluster represents the gene’s expression profile.

To determine differentially expressed genes, DESeq’s nbinom test function was used to test the significance of the differences between the base means of the two conditions (e.g.,washed vs unwashed, colostral vs transitional, etc.). Differences with *p*<0.05 after adjustment for multiple hypothesis testing (method = “BH”) were deemed statistically significant.

We determined biological functions of gene sets of interest using the Functional Annotation Clustering tool within DAVID Bioinformatics Resources 6.7 [53], [54]. All Ensembl IDs were used as the background gene list. Enriched annotations associated with *p*<0.05 after adjustment for multiple hypothesis testing (method = “Benjamini”) were deemed statistically significant. Annotation clusters were considered significant only if they contained statistically enriched annotations.

### Confirmation of RNA-Seq Results with qPCR

We confirmed the RNA-Seq results for genes of interest with TaqMan qPCR. Total RNA from four mature milk fat fractions were synthesized into cDNA using the SuperScript® III First-strand Synthesis SuperMix (Invitrogen, Carlsbad, CA) according to manufacturer’s instructions. Samples were then ethanol precipitated to enhance purity of DNA. Genes of interest (n = 15) were validated using qPCR with Custom TaqMan® Array Plates in a 96-well format (Applied Biosystems, Carlsbad, CA). Details on the primer probes sets for these genes are provided in **Dataset S5**. Reactions consisted of 12.5 µL TaqMan® 2× Gene Expression Master Mix, 7.5 µL H_2_O, and 5 µL (12.5 ng) template, for a total volume of 25 µL per well. All samples were assayed in duplicate for each gene of interest. Each plate was run on the Applied Biosystems StepOnePlus™ using standard settings (cycling program included 10 min incubation at 95°C followed by 40 cycles of 15 sec at 95°C and 1 min at 60°C). Data were assessed from each assay using the StepOnePlus™ software tool v2.2.

Following the approach of Bionaz and Loor [55], cycle thresholds (Ct) were normalized to the geometric mean of three endogenous genes. We selected SSH3, ACTB, and RPS18 to comprise our endogenous gene set. The first (SSH3) was selected from the RNA-sequenced transcriptome based on expression stability across samples (coefficient of variation, 10%) and the availability of a reliable primer-probe set. SSH3 is involved in the regulation of the actin cytoskeleton of cells. ACTB and RPS18 are commonly used as control genes. Both demonstrated reasonable stability across samples (coefficient of variation, 18% and 24%, respectively).

To compare RNA-Seq results with qPCR for each gene of interest, RNASeq FPKM values were normalized to the FPKM geometric mean of the endogenous gene set (SSH3, ACTB, RPS18). We then determined the Pearson linear correlation coefficient (on a log scale) between the normalized qPCR-Ct and FPKM means across the 15 genes of interest. In this analysis, each gene is an observation, with two variables per gene: qPCR-Ct and FPKM. The values for these two variables, per observation, are the (log) normalized qPCR-Ct and FPKM means of the 4 individual samples.

## Supporting Information

Figure S1 **Milk fat layer under the fluorescent microscope.** Fresh milk fat layer solids were fixed on a slide with a cytospin, stained with DAPI, and examined on a Zeiss florescent microscope for blue-fluorescing nuclei as evidence of intact cell infiltration (in contrast, mammary epithelial cell remnants trapped in secreted fat globules will not contain nuclei). Non-nuclei cell debris stain green. **Panel A**: Colostrum milk fat layer at 20× was heavily infiltrated with intact cells. **Panel B**: Mature milk fat layer at 40× was also infiltrated with intact cells, but to a much lesser extent.
(TIFF)

Figure S2 **Mean (±SEM) FPKM for leukocyte cell markers.** Regardless of washing protocol, mature samples showed virtually no evidence of leukocyte specific gene expression; however, colostral–and transitional samples to a much lesser extent–show at least some evidence of leukocyte cell gene expression. In contrast to the other leukocyte cell markers in this figure, CD33 (a cell marker heavily expressed in immature stem cells) expression is not significantly different between colostral and mature lactation, providing evidence of human milk being a source of stem cells. Footnotes: Col = Colostral, Tra = Transitional, and Mat = Mature stages of lactation; *p<0.05, Colostral versus Transitional; +*p*<0.05, Transitional versus Mature.
(TIFF)

Figure S3 **Cluster dendogram of all RNA-sequenced samples.** Clustering analysis produced a dendogram in which lactation stage, as biochemically defined by Na:K ratio, emerged as the greatest overall difference in transcriptomes across samples.
(TIFF)

Table S1 **Summary of milk fat globule RNA quality by stage of lactation and processing method.**
(DOCX)

Table S2 **Summary of maternal and sample characteristics for all RNA sequenced samples.**
(DOCX)

Dataset S1 **Gene sets and their FPKM values, identified by lactation stage and processing method.**
(XLSX)

Dataset S2 **Enrichment of functional annotations for the top ten percent of genes expressed in each stage of lactation.**
(XLSX)

Dataset S3 **Significant KEGG pathways and Biological Process Gene Ontology Terms for each of the eight identified cluster trajectories.**
(XLSX)

Dataset S4 **Insulin signaling and lactose synthesis pathway gene sets, their FPKM values at each lactation stage, and whether upregulated, downregulated or unchanged between stages.**
(XLSX)

Dataset S5 **Details on primer probes sets for PCR assay.**
(XLSX)
