# Supplementary figures and images for: RNA Sequencing of the Human Milk Fat Layer Transcriptome Reveals Distinct Gene Expression Profiles at Three Stages of Lactation
Source: PLoS One. 2013 Jul 5;8(7):e67531. doi: 10.1371/journal.pone.0067531 (PMC3702532; doi:10.1371/journal.pone.0067531)

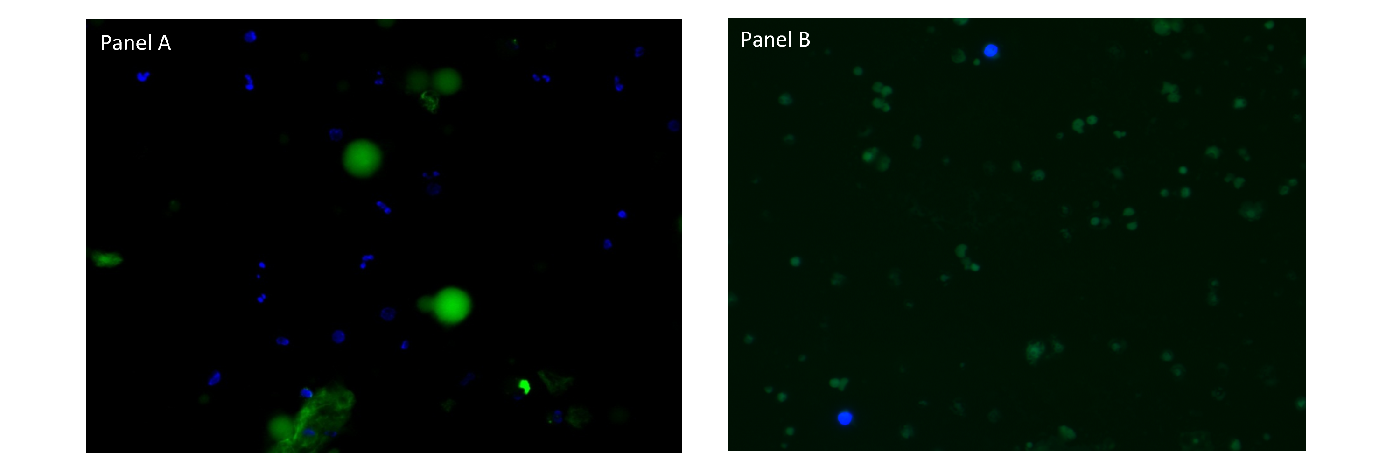

Supplement: Figure S1 — Milk fat layer under the fluorescent microscope. Fresh milk fat layer solids were fixed on a slide with a cytospin, stained with DAPI, and examined on a Zeiss florescent microscope for blue-fluorescing nuclei as evidence of intact cell infiltration (in contrast, mammary epithelial cell remnants trapped in secreted fat globules will not contain nuclei). Non-nuclei cell debris stain green. Panel A: Colostrum milk fat layer at 20× was heavily infiltrated with intact cells. Panel B: Mature milk fat layer at 40× was also infiltrated with intact cells, but to a much lesser extent. (TIFF) [file pone.0067531.s001.tif]

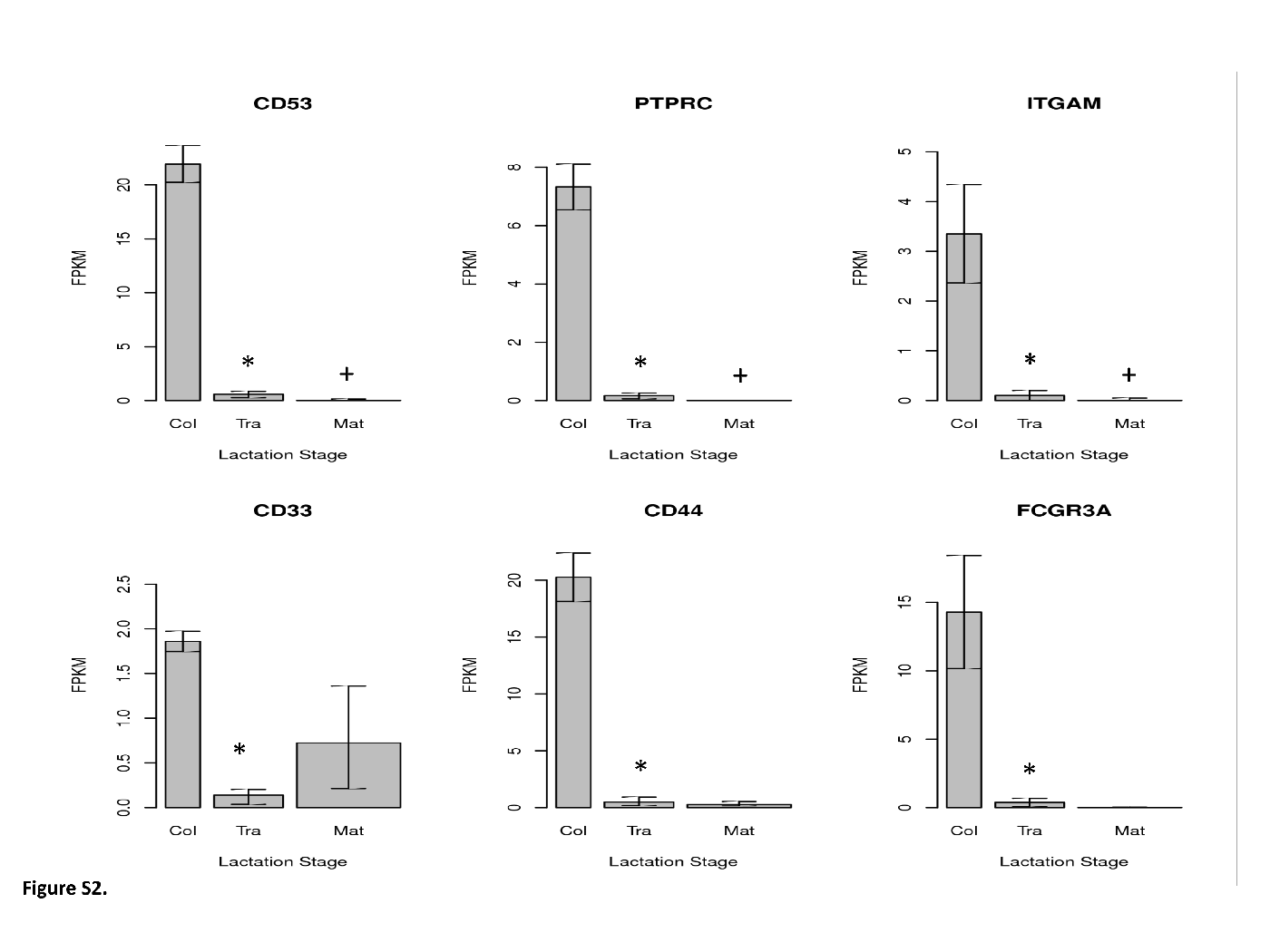

Supplement: Figure S2 — Mean (±SEM) FPKM for leukocyte cell markers. Regardless of washing protocol, mature samples showed virtually no evidence of leukocyte specific gene expression; however, colostral–and transitional samples to a much lesser extent–show at least some evidence of leukocyte cell gene expression. In contrast to the other leukocyte cell markers in this figure, CD33 (a cell marker heavily expressed in immature stem cells) expression is not significantly different between colostral and mature lactation, providing evidence of human milk being a source of stem cells. Footnotes: Col = Colostral, Tra = Transitional, and Mat = Mature stages of lactation; *p<0.05, Colostral versus Transitional; +p<0.05, Transitional versus Mature. (TIFF) [file pone.0067531.s002.tif]

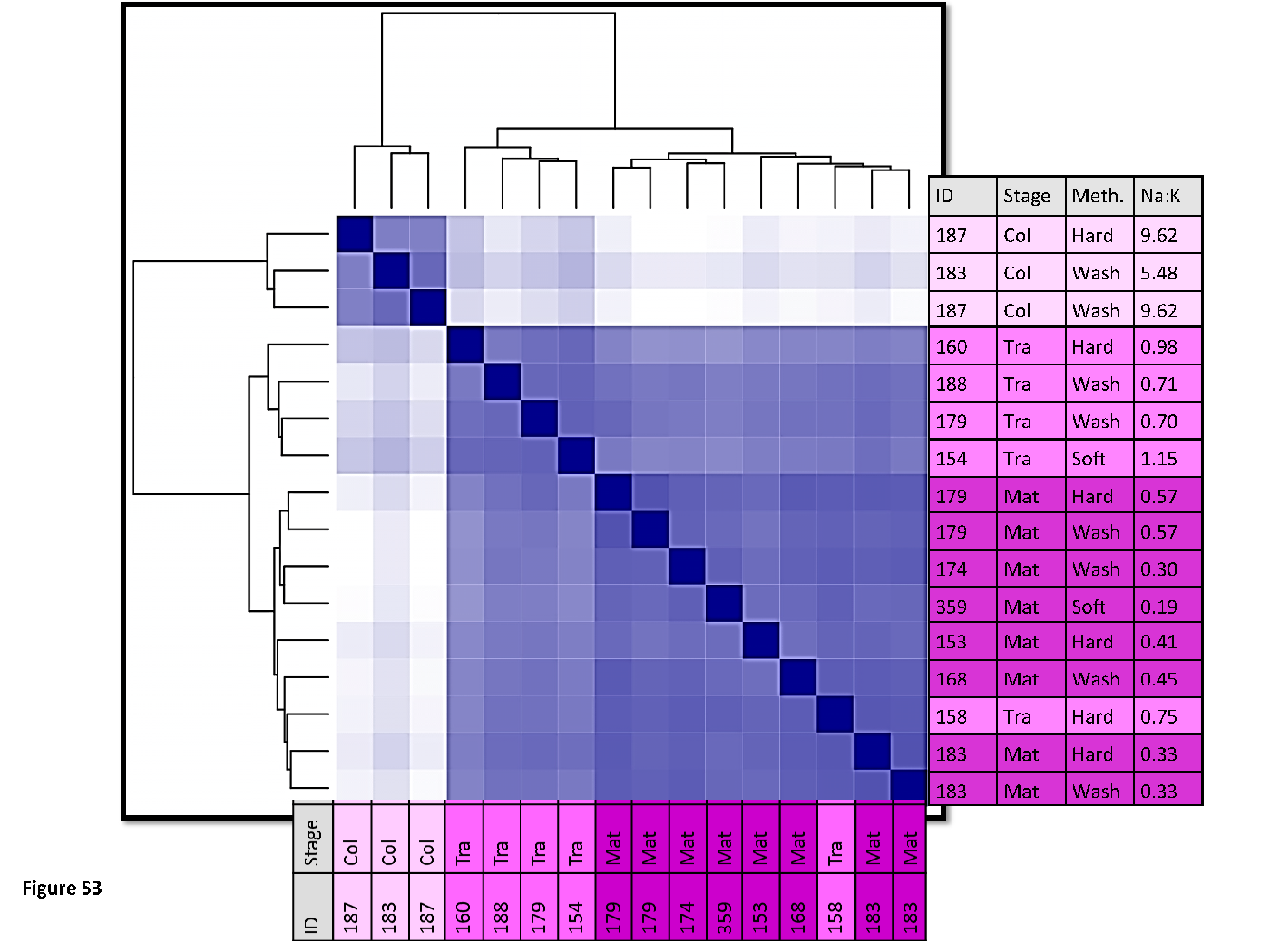

Supplement: Figure S3 — Cluster dendogram of all RNA-sequenced samples. Clustering analysis produced a dendogram in which lactation stage, as biochemically defined by Na:K ratio, emerged as the greatest overall difference in transcriptomes across samples. (TIFF) [file pone.0067531.s003.tif]
