# Supplementary material for: RNA Sequencing of the Human Milk Fat Layer Transcriptome Reveals Distinct Gene Expression Profiles at Three Stages of Lactation
Source: PLoS One. 2013 Jul 5;8(7):e67531. doi: 10.1371/journal.pone.0067531 (PMC3702532; doi:10.1371/journal.pone.0067531)
Supplement: Table S1 — Summary of milk fat globule RNA quality by stage of lactation and processing method. (DOCX) [file pone.0067531.s004.docx]

**Table S1. Summary of milk fat globule RNA quality by stage of lactation and processing method for all samples processed (N=55)**

| **Stage of lactation** | **Timing of sample collection**  **Median (min-max)** | **Na:K ratio**  **Median (min-max)** | **Processing Method^a^** | **Suitable for sequencing^b^**  **No. (%)*** |
| --- | --- | --- | --- | --- |
| Day 2, Colostral^c^ | 45.8 (37.0-64.8) hours | 3.44 (2.10-9.62) | Soft | 0 of 1 (0) |
|  |  |  | Hard | 1 of 4 (25) |
|  |  |  | Washed | 2 of 6 (33) |
|  |  |  | **Overall** | **3 of 11 (27)** |
| Day 2, Transitional^d^ | 46.5 (38.9-74.2) hours | 1.09 (0.42-1.86) | Soft | 1 of 2 (50) |
|  |  |  | Hard | 4 of 5 (80) |
|  |  |  | Washed | 4 of 8 (50) |
|  |  |  | **Overall** | **9 of 15 (60)** |
| Mature^e^ | 45 (24-368) days | 0.35 (0.19-0.89) | Soft | 3 of 5 (60) |
|  |  |  | Hard^e^ | 7 of 9 (77) |
|  |  |  | Washed | 11 of 15 (73) |
|  |  |  | **Overall** | **21 of 29 (72)** |

*Proportion suitable for sequencing by stage of lactation, Chi-square=6.7, *p*=0.03

^a^ See Methods for an explanation of milk sample processing categories

^b^ Suitable for sequencing defined as RIN > 7.0 and RNA > 10 ng/uL (the latter excluded 4 Colostrum, 1 Transitional, and 1 Mature)

^c,d^ Lactation stage biochemically defined as colostral, Na:K > 2.0; and transitional, Na:K < 2.0
