# Supplementary material for: RNA Sequencing of the Human Milk Fat Layer Transcriptome Reveals Distinct Gene Expression Profiles at Three Stages of Lactation
Source: PLoS One. 2013 Jul 5;8(7):e67531. doi: 10.1371/journal.pone.0067531 (PMC3702532; doi:10.1371/journal.pone.0067531)
Supplement: Table S2 — Summary of maternal and sample characteristics for all RNA sequenced samples. (DOCX) [file pone.0067531.s005.docx]

| Participant | | | | | | | Day 2 Milk Sample | | | | | Mature Milk Sample | | | | | Comment |
| --- | --- | --- | --- | --- | --- | --- | --- | --- | --- | --- | --- | --- | --- | --- | --- | --- | --- |
| ID | Age,  Parity | BMI | Breast full hour | Glucose Tolerance | | | Hour PP | Na:K | BF % | RIN, Fat Globule RNA | | Day PP | Na:K | BF % | RIN, Fat Globule RNA | |  |
|  |  |  |  | Basal | SEC | SEN |  |  |  | UW | W2 |  |  |  | UW | W2 |  |
| 187 | 30, P | 26.7 | 59 | -- | -- | -- | 41 | 9.62 | 100 | 8.0 **^E^** | **8.1^C^** | -- | -- | -- | -- | -- |  |
| 183 | 38, M | 27.5 | 74 | 6.7, 90 | n/a | n/a | 52 | 5.48 | 100 | -- | **8.7^C^** | 24 | 0.33 | 100 | 9.0 **^E^** | **8.9^M^** | Milk supply concern initially, but resolved |
| 154 | 30, P | 31.3 | 63 | -- | -- | -- | 49 | 1.15 | 100 | **9.7^T^** | -- | -- | -- | -- | -- | -- |  |
| 160 | 26, P | 22.2 | 69 | -- | -- | -- | 56 | 0.98 | 100 | **7.6^T^** | -- | -- | -- | -- | -- | -- |  |
| 158 | 23, P | 42.0 | 34 | 9.2,  86 | Hi,  3.4 | Lo,  4.3 | 44 | 0.75 | 100 | 8.6**^E^** | -- | -- | -- | -- | -- | -- | Severe mastitis day 11 |
| 188 | 33, M | 27.1 | 50 | -- | -- | -- | 52 | 0.71 | 100 | -- | **8.9^T^** | -- | -- | -- | -- | -- |  |
| 179 | 36, M | 28.0 | 33 | 5.7, 82 | Hi, 2.1 | Hi, 6.7 | 39 | 0.70 | 100 | -- | **8.8^T^** | 35 | 0.57 | 100 | 8.3 **^E^** | **8.3^M^** | Mild (maternal) cold on collection day |
| 359 | 28, P | -- | -- | -- | -- | -- | -- | -- | -- | -- | -- | 130 | 0.19 | 100 | **8.3^M^** | -- | Protocol-II |
| 174 | 32, M | 41.3 | 34 | 9.0,  92 | Hi, 1.9 | Hi, 4.7 | -- | -- | -- | -- | -- | 33 | 0.30 | 100 | -- | **9.0^M^** |  |
| 153 | 33, P | 36.0 | 77 | 9.8,  97 | Lo, 1.5 | Lo, 3.5 | -- | -- | -- | -- | -- | 40 | 0.41 | 58 | **7.7^M^** | -- | Milk supply concern, borderline GDM |
| 168 | 34, M | 33.1 | 71 | 14.1,  105 | Lo, 1.7 | Lo, 2.6 | -- | -- | -- | -- | -- | 45 | 0.45 | 94 | -- | **9.0^M^** | Milk supply concern |

**Table S2. Sequenced samples, summary of maternal and sample characteristics***

*****Abbreviations: **BMI**=Body Mass Index (early postpartum); **Breast full hour**=Postpartum onset of notable breast fullness based on maternal report; **Basal**=Fasting insulin (uU/mL, median=9.0, range=3.3-15.5, based on full sample of 12 follow-up participants), Fasting glucose (mg/100 mL, median=88, range=76-105, based on full sample of 12 follow-up participants); **SEC**= Insulin Secretion, estimated pancreatic beta cell response to glucose load, ISSI-2 calculation [Retnarkian, 2010] (**Lo/Hi**=below/above median based on full sample of 12 follow-up participants, median=1.9, range=1.3-3.8); **SEN**=Insulin Sensitivity, estimated whole-body insulin sensitivity, ISOGTT calculation [Matsuda, 1999] (**Lo/Hi**=below/above median based on full sample of 12 follow-up participants, median=4.5, range=2.6-12.3); **n/a**=post-time 0 blood samples hemolyzed, rendering insulin analysis invalid; **PP**= Postpartum; **Na:K**=Sodium to potassium ratio in aqueous fraction of sample; **BF %** = Breastfeeding as a percent of all feeds; **RIN**=RNA integrity number; **UW**=Unwashed processing method (all were hard spin except 154 and 359); **W2**=Washed twice method; **P**=Primiparous; **M**=Multiparous; ( **--** ) = Data not available; **GDM**=Gestational Diabetes Mellitus

**^C^**Colostrum (N=2), **^T^**Transitional (N=4), and **^M^**Mature (N=6) samples included in final bioinformatics analysis; **^E^**Excluded from final bioinformatics analysis either because unwashed partner of unwashed/washed split (N=1 Transitional, N=2 Mature) or mastitis (N=1)
